# An informatics strategy for cancer care

**DOI:** 10.2349/biij.4.3.e35

**Published:** 2008-07-01

**Authors:** J Wright, A Shogan, J McCune, S Stevens

**Affiliations:** UPMC Cancer Centers, Information Technology Department, Pittsburgh, Pennsylvania, United States

**Keywords:** Oncology informatics, informatics strategy

## Abstract

Whether transitioning from paper to electronic records or attempting to leverage data from existing systems for outcome studies, oncology practices face many challenges in defining and executing an informatics strategy. With the increasing costs of oncology treatments and expected changes in reimbursement rules, including requirements for evidence that supports physician decisions, it will become essential to collect data on treatment decisions and treatment efficacy to run a successful program. This study evaluates the current state of informatics systems available for use in oncology programs and focuses on developing an informatics strategy to meet the challenges introduced by expected changes in reimbursement rules and in medical and information technologies.

## INTRODUCTION

Whether transitioning from paper to electronic records or attempting to leverage data from existing systems for outcome studies, oncology practices face many challenges in defining and executing an informatics strategy. With the increasing cost of oncology treatments and expected changes in reimbursement rules, including requirements for evidence that supports physician decisions, it will become essential to collect data on treatment decisions and treatment efficacy to run a successful program.

The ability to measure efficacy of care and to identify actionable areas for improvement has proven evasive in the specialty healthcare software market. Oncology electronic medical record systems (EMRs) are largely workflow-centric, focusing mainly on a series of tasks, but not on facilitating real-time decision-making and comprehensive outcomes collection.

The treatment of cancer requires long-term patient management, which often spans decades. Each patient case can have a significant amount of information ranging from test results to medical administration records, clinical evaluation notes, as well as insurance and billing records. As a result, there is a wealth of data collected over time on the diagnosis, treatment and outcomes, but most often this data resides only on paper. Transitioning from paper to an electronic medical record system or even supplementing paper records with an EMR involves significant time and manpower for data entry, training and maintenance.

Oncology information systems must support the collection of relevant data for today’s medical practices, while also allowing for the integration of future developments in the medical field. Evolving technology will result in more personalised medicine with rapid feedback mechanisms. It is important for an electronic oncology system to have the ability to interface with these technologies in order to diagnose response to treatment and incorporate new knowledge into standards of care when appropriate. Interoperability with new clinical tools and systems will be critical in maintaining accurate and complete patient records.

### Economics

Performance will be the key economic factor for oncologists and oncology practices. As the medical field becomes increasingly focused on pay-for-performance, it is inevitable that hospitals, insurance companies and other organisations will increase their quality of care requirements. Business administrators must consider the following:

- Fee-For-Service (FFS) reimbursement programs are being replaced by value-based systems such as Pay-For-Performance (P4P) in the U.S. and by other performance-based systems globally [1, 2].
- P4P will be based on quality indicators that are more meaningful and derived from specific diagnostic tests.
- Measuring response to treatment and toxicity will be critical to ensure an accurate performance metric for reimbursement [1, 4].
- Outcomes and performance indicators will be based on current data and compared across the industry.
- Potential benefits will appear from sharing highly effective treatment plans with other physicians and practices.
- Performance-based programs will influence standards of care that the industry can use to set prices and reimbursement rates.

As performance becomes the key economic factor for the physician, it is critical for oncology systems to generate reports on cost performance and to facilitate outcome studies that show quality performance based on treatment efficacy and toxicities. Practices will be ‘rewarded’ for demonstrating quality and efficiency in care delivery [1-4]. ‘Normative’ economics will steer decision-making to consider the cost/value ratio of a procedure in relation to potential outcome. Insurance companies will require detailed evidence of specific conditions before approving procedures and drugs. Segmenting a provider’s patients into logical groupings based on condition/progress will be essential when approximating financials, measuring outcomes and comparing performance. Biotechnology, pharmaceutical and medical device vendors will be forced to price their products based on evidentiary positive results.

This study will evaluate the current state of informatics systems available for use in oncology programs. It is expected that the abilities of the currently available systems will not be fully aligned with the primary needs of the oncology field. The functional capabilities and problems with oncology informatics today will be compared to the needs of the stakeholders and, if applicable, suggestions to improve the development of electronic oncology systems will be made. In particular, suggestions will focus on developing an informatics strategy to meet the challenges introduced by expected changes in reimbursement rules and in medical and information technologies.

## A TOP-DOWN APPROACH

### Oncology Informatics Stakeholders

**Figure 1:**
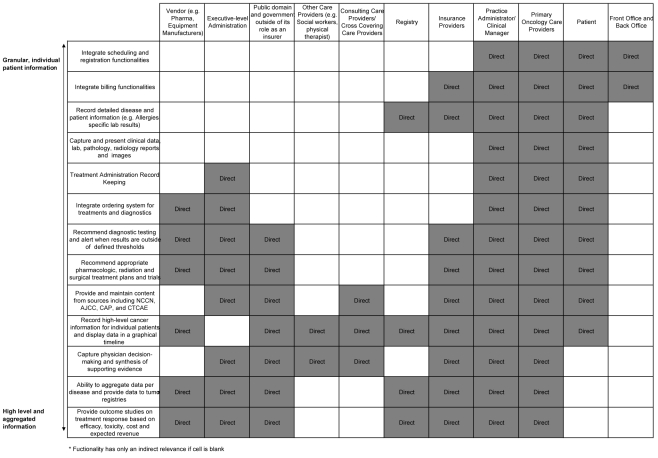
Relevance of Functionality to Stakeholder.

A review of oncology informatics must start by looking at the information needs of the various stakeholders who serve to gain from a more efficient method of tracking patient information. Care providers, administrators, insurance companies, and patients are just a few examples of these stakeholders. Data requirements range from individual patient records to aggregate sets of data for analysis and reporting. As shown in Figure 1, the data and functionality most relevant to the majority of stakeholders involves the synthesis and aggregation of information, not those that involve highly detailed individual patient data.

Care providers have an interest in all clinical data associated with the patient. Clinicians and administrators need a way to track a patient’s overall status, including treatment plan, response to treatment, toxicities associated with treatment, and information specific to clinical trials. Physicians rely on a patient’s data for diagnosis and care decisions. Administrators look at data aggregates to assess quality of care and compliance to standards.

**Figure 2:**
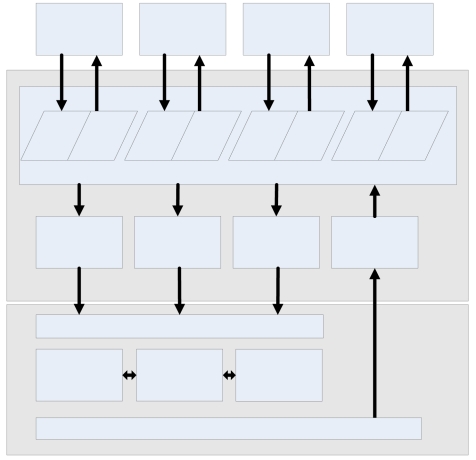
A Top-Down Approach.

As shown in Figure 2, external entities including insurance companies, drug and equipment vendors, tumour registries and government agencies provide influence related to costs, reimbursements, treatment efficacy, and diagnostic efficacy, all of which may be used by the oncology practice as the basis for decision-making. Clinical and business knowledge can be shared with external entities to leverage contractual arrangements, benefit public domain medical information, and improve quality of care. These stakeholders can then use this knowledge to fine-tune costs, rates, and their contractual commitments.

Research and registry department stakeholders are in need of tools to retrieve, synthesise, and aggregate information from patient charts for registry reporting and outcome studies. When done manually, this is a labour-intensive and lengthy process that can take several months. Clinical systems available today do not provide tools to meet business requirements for registry or outcome studies.

## INFORMATICS ADDRESSING THE NEEDS OF TODAY’S ONCOLOGY PRACTICES

In today’s healthcare information technology environment, there are a number of systems that focus on specific tasks. Some systems are suitable for any type of ambulatory setting and thus can be used in oncology practices. However, only a small number of systems have been developed specifically for the oncology space. An example of the latter would be the systems used with oncology treatment equipment that rely on integrated software to control and support radiotherapy and infusion administration.

Informatics technologies supporting the oncology business practice and patient medical records fall into the following categories:

### Practice Management Systems

Practice Management systems, providing patient registration, scheduling and billing functionality, are a logical first step in producing an electronic medical record. The foundation of the patient record is built by collecting patient demographics and insurance information, and tracking patient visits and services for billing purposes. Billing functionality supports the labour-intensive tasks and expensive medical equipment usage, and ensures the accuracy required to generate revenue for the business. There are several systems that have been designed for use in any type of medical practice and can, therefore, be applied to oncology practices. These Practice Management systems continue to mature.

### Electronic Medical Record Systems

Electronic Medical Record (EMR) systems are typically seen as the next step in moving toward electronic patient records. EMR systems facilitate workflow in the office, provide forms and functionality for clinical documentation and visit management, track treatment administration, and support order entry. EMRs usually include automated dosage validation, and drug interaction and allergy alerts when placing orders. Compared to practice management systems, there are fewer oncology EMR systems from which to choose. Some EMR vendors are in the early stages of integrating records for medical oncology and radiation oncology, but for the most part, EMR systems are designed to meet the needs of one modality or the other. Built for ambulatory settings, there is little or no tracking of a patient’s inpatient care.

### Clinical Decision Support Systems

The importance of clinical decision support systems that guide patient care and ensure patient safety is well-recognised, although such systems are still in the very early stage of implementation for the oncology practice. At its best, the clinical decision support system will focus on providing diagnostic test results to inform physicians during decision-making and will document the evidence used to support decisions. Tools will allow for the analysis of discrete evidence to provide better, more cost-effective guidelines for diagnostic testing. Rules engines will be responsible for presenting treatment guidelines to physicians at the point of care based on disease and stage diagnosis, relevant patient characteristics, and treatment history. Alerts will warn the physician when a patient co-morbidity puts the patient at greater risk for toxicities associated with an intervention. Knowledge obtained from studies of treatment efficacy and toxicities will be used to recommend and improve treatments. Industry standards from organisations such as National Comprehensive Cancer Network (NCCN), American Joint Committee on Cancer (AJCC), College of American Pathologists (CAP), and National Cancer Institute (NCI) will be incorporated for decision support. Physicians will be guided to follow site-defined standards of care. Cost and reimbursement of treatments and testing will be tracked against studies of treatment and testing efficacy, allowing for improvements to the financial health of the organisation and for use in facilitating contract negotiations.

### Clinical Standards of Care Authoring

Establishing intervention standards of care for an oncology organisation is facilitated with tools for authoring, making revisions, providing references and citations, and managing the approval process. The authoring tool allows interventions and treatment plans to be tied to specific cancer profiles and to be prioritised based on outcome and economic factors. This tool also facilitates the creation and maintenance of order sets associated with interventions. Working together with a decision support system, internal treatment guidelines can be recommended when appropriate for the patient, disease, and stage. Analytical tools that provide costs and expected reimbursements for treatments can assist with the authoring. Reports on compliance, based on data from decision support, can help to identify issues and improve standards. Although authoring systems do exist, systems designed specifically for authoring oncology treatment plans based on cancer diagnosis and stage are not found in today’s market.

### Outcome Studies

By tracking the response of the disease and the patient to treatment, outcome studies are made possible. Collecting data specific to the diagnosis of disease and stage, monitoring the status of disease with a minimal set of relevant questions, recording the use of interventions, the response of the disease to treatment, and the toxicities experienced by the patient, as well as documenting evidence that supports the diagnosis of disease, stage and response creates a rich knowledge-base that fosters an environment of continual improvement based on acquired evidence. Knowledge will be the key competitive differentiator for physicians. Guideline collaboratives will evolve, allowing smaller practices to remain competitive. Software will facilitate the ability to tie guidelines and protocols to specific condition profiles and to prioritise their use based on outcome and economic factors. Practices will be positioned to respond to payer priorities for highly effective diagnostics and treatments.

### Clinical Trial Management

According to a statement on the caBIG website:

“At present, there is no standardized, uniform system for clinical trial management, and no central registry of all cancer-related clinical trials. Moreover, only a small number of cancer patients actually participate in clinical trials of experimental therapies. ‘Legacy,’ or paper-based, information systems for clinical trials are slow and inefficient for the collection, analysis and sharing of data, resulting in unnecessary work delays, duplication of effort, human errors in data entry, and missed opportunities for data mining and secondary use of the data.” [5]

As more software is developed in this area, it will be important to look for interoperability, discrete eligibility requirements, and financial management functionality.

### Ancillary Systems

Oncology is supported by specialised and mature ancillary systems that are used throughout healthcare, including:

- Radiographic, pathologic, and lab diagnostics systems.
- Drug dispensing and inventory management systems.
- Dictation systems that provide transcribed notes to EMR systems.

### Expectations

As ambulatory services grow, the healthcare field is beginning to see growth in software development for ambulatory settings. Gartner, the global leader in providing accurate and current research for the information technology industry, predicts that there will be mainstream adoption of ambulatory EMR systems within two to five years [6]. The number of practice management systems and EMR systems aimed at either radiation oncology or medical oncology has grown in recent years. As these begin to mature, we will start to see IT vendors’ focus shifting towards the integration of medical, radiation, and surgical records, interoperability of EMR and diagnostic systems, as well as development of clinical decision support systems and data repositories that support research and outcome studies. Strategies will be developed to address the implementation and workflow issues presented by stand-alone practices, integrated oncology practice networks, and large academic hospital-based centres.

## PROBLEMS WITH EXISTING ONCOLOGY SYSTEMS

### Interoperability

System interoperability is required to ensure critical patient data is shared across all healthcare systems. Electronic medical systems are often designed for one part of the oncology care team. With limited sharing of information, there can be unnecessary repetition of costly and potentially painful diagnostic procedures. Areas in need of enhanced interoperability include:

- Integrated views across care settings including medical, radiation, and surgical oncology in both inpatient and ambulatory settings - it will be imperative for software to facilitate a collaborative care approach.
- Clinical, pathologic, and radiographic diagnostic results visibility in oncology systems as needed and ability to add new diagnostic technologies as they emerge.
- Integration of clinical and financial business systems.
- Patient identification technologies.
- System upload/download capability to/from CDs or cards embedded with computer chips.
- Provision of patient identity and order data to infusion pumps, pharmacy dispensers, and radiation therapy planning systems for validation, and in turn, treatment data sent back for treatment administration records.
- Information exchange among practices within an organisation and with external healthcare organisations including tumour registries and insurance providers.
- Standards for clinical terminology and ontology-based semantic interoperability allowing information exchange.
- Caregiver access to patient history, progress, current status, medical necessity, and continuity of care data in near-real-time.
- Physician remote access to patient records.
- Patient portals for education, financial account management, scheduling, and other communication with providers.

### Usability Challenges

One of the greatest challenges facing IT vendors today is integrating systems into the physician’s busy workflow without requiring additional time from the physician. Many hardware solutions including handhelds (PDAs), portables (tablets/slates), and desktops in the exam room are readily available. However, issues regarding patient privacy, time for login, patient search, data entry, and the impact on the physician–patient interaction are difficult ones that need to be addressed practice-by-practice, if not physician-by-physician. Changing the culture from paper to electronic notation will require physician ‘champions’ to overcome the barriers. In the end, hardware requirements and staffing for maintenance of hardware and software are often beyond what a stand-alone oncology practice can reasonably manage.

### Data Limitations

To be effective, data collection and storage must be carefully designed. Some problems that oncology system users currently face are:

- Discrete data specific to oncology isn’t captured in a way that can be used for reporting, trending, or outcome studies.
- Physician decision-making process is documented as textual notes.
- Systems aren't adaptable for new technologies and knowledge content (e.g. treatment guideline updates).
- High percentage of data presented to physician is not relevant to oncology treatment .
- There is no comprehensive summary of the status of disease and patient toxicities.
- Registry staff must manually synthesise data in charts for tumour registry.
- Response to treatment isn’t captured.
- Standards of care aren’t promoted with recommended treatment options.
- A treatment feedback loop is not supported – for example: diagnosis/prognosis, presentation of treatment standards for selection, treatment administration, monitoring/adjustment, recording of response, follow-up visit, reporting / feedback to improve standards of care.
- Summary of care doesn’t clearly represent an overview of oncology-specific events or timeline with inflection points.
- Tracking of treatment efficacy versus costs and reimbursement is poorly facilitated.
- High cost of data entry for detailed research/registry data.

### Keeping Pace with New Technologies

Electronic oncology systems must remain flexible and adaptable to allow for new technology and regulation changes. Upcoming changes are expected to include:

#### Diagnostic Techniques

Systems must be flexible and able to adapt to the growing use of molecular imaging and data such as DNA sequencing, levels of genetic expression, biomarkers, and protein structure for diagnosis and treatment [7, 8]. The ability to look at molecular data together with clinical, pathologic and radiographic data will provide a wealth of research material and evidence supporting diagnosis and treatment decisions.

Molecular imaging, proteomics and the use of biomarkers, and the study of temperature, gravitational properties and density changes in tumours are likely to make important contributions to diagnostic data. These technologies will continue to evolve and will be used to identify and monitor response to treatment more effectively.

#### Treatment Technology

Support for the administration of collaborative treatment plans, including chemotherapy, immunotherapy, radiation therapy, and surgery is currently still very much a need in cancer care. Stem cell transplants, cryotherapy, tomotherapy, proton therapy, photodynamic therapy, and signal transduction therapy are examples of interventions that are not captured electronically in existing systems. As new techniques are developed or become more commonly used, these too must be incorporated into systems to allow for a comprehensive look at patient treatment history and care. Support for site configurable treatment protocols and rules for each type of intervention will also be needed. For order set construction, the use of drugs, herbs, massage, acupuncture, music therapy, and diet to manage side effects must be supported.

#### Computer Technology and Human Computer Interaction (HCI)

Current initiatives in hardware, communication, and application software development will help to make the transition to the use of electronic systems a more natural fit with physician thought-flow and practice workflows. Wireless enabled user interfaces, portable devices, and touchpad screens can help with usability. Virtualised hardware, operating systems, storage, and centrally hosted software delivered as a service (SAAS) can be used to drive implementation and maintenance costs down. Natural Language Processing (NLP) will be used to streamline data input by saving physician dictation directly to disk. Work is being done to structure data contained in pathology and radiology reports, which will provide a comprehensive diagnosis that can be viewed at a very granular level for analysis and studies. Service Oriented Architectures (SOA) and modularised application components that are interchangeable and reusable across the health enterprise will improve information exchange and will facilitate a build-as-you-go, prioritised approach to system procurement. Gartner predicts that mainstream adoption of Natural Language Processing will occur in 2 to 5 years and that adoption of SOA for application integration in healthcare will occur in 5 to 10 years, whereas semantic interoperability will likely take more than 10 years to become widely adopted [9, 10].

### Global Considerations

Software products and information resources must be designed to meet requirements for customers in any country or region. Oncology centres that expand to include practices in multiple countries will need to have the ability to configure settings that are appropriate for each practice. For example, configuration will be required to define units of measure; currency formats; date and time formats; data element types and lengths; language options for displays and keyboard; language options for speech recognition and handwriting recognition if these features are supported by the system; content (e.g. diagnostic and billing codes, disease and stage classifications that are suitable for the local population); rules such as. reimbursement rules and tumour registry rules; local laws and regulations (e.g. some countries require that servers holding patient data must be physically located in the country of the treating facility); availability of treatments and diagnostics technologies; and implementation strategies (e.g. the impact of a change to regional settings at the operating system level on other applications).

In developing countries, lack of infrastructure, communication services, and access to research findings available on the Internet can impede progress and affect quality of care [11]. Internet access alone can provide many resources to help oncologists throughout the world. For example, clinical practice guidelines from the National Comprehensive Cancer Network, Inc (NCCN) are currently available in English, Spanish, Chinese, and Japanese [12, 13]. Guides for developing countries on medical records, electronic health records, and improving data quality are provided on a website created for the World Health Organization Regional Office for the Western Pacific [14]. Quality initiatives are promoted on websites such as those described on the website of the Taipei Branch of Bureau of National Health Insurance [15]. Information about the Quality and Outcomes Framework (QOF) in England and the Quality Management and Analysis System that supports QOF are available on a site that provides online help and training information [16]. The PubMed Databases and search engines available on the website of the National Center for Biotechnology Information (NCBI) provide a wealth of research findings. An online search returns 2328 citations when searching for “oncology in Asia” (in quotes). Adding “and breast” (without quotes) will narrow down the search to 289 results [17].

## IN SEARCH OF THE IDEAL ONCOLOGY INFORMATION SYSTEM

In a typical medical record (EMR or paper record) information about the patient’s cancer is obtained by extracting data from office notes, lab results, X-ray reports, and other tests. Even though the clinician has all of the above information, he or she is still required to synthesise the data. Frequently, a summary of the synthesis is entered as free-form text in the office note. While the information contained in the note is critical, the note itself is often long and complicated.

Additionally, data from administrative, financial and billing systems are not connected with the clinical data in any meaningful way. In the newly developing systems of insurance, oncology practices will only be reimbursed for a drug (often a very expensive drug) if it is approved for a particular cancer, a particular stage of that cancer, and sometimes, even a subset of the cancer that expresses a certain laboratory-tested phenotype or genotype. There is currently no easy way for administrative and billing personnel to verify this. Furthermore, the shift to pay-for-performance reimbursement will require an institution to have virtual real-time access to intervention and response data for the various diseases. Capitated insurance contracts will require knowing how much is spent in pharmacologic therapy, radiation therapy, or surgery to treat a particular stage of disease.

An effective electronic oncology system will be able to address these issues by: 1) promoting standards of care; 2) supporting and capturing the physician’s decision-making process when diagnosing disease, stage and status of disease, when deciding how to treat the disease, and when determining response to treatment; 3) establishing relationships of treatment to disease stage and status diagnosis, outcomes, costs, and revenue; 4) capturing outcomes and performance metrics; and 5) associating diagnostic results as evidence that supports the diagnosis of disease, stage, and outcomes.

The development of healthcare systems designed for decision support and outcome studies generally follows the development of EMR systems. For oncology needs however, it is worth considering a different approach. Once a Practice Management System has been implemented, thought should be given to planning for clinical decision support and outcome studies as the next strategic step. With access to a summary of the patient’s full oncology treatment history and records of response to treatment, the oncologist will have the support needed to make individualised treatment decisions. With the support of standards of care that are based on the disease, stage, patient and disease characteristics, and evidence of successful interventions, the oncologist can be guided to treat effectively and consistently in all situations. The innate value of a decision support system will be derived from facilitating, aiding and capturing the physician’s decision-making process through the continuum of the patient’s care. Providing the physician with guidance and a concise view into the realm of relevant patient disease information will result in clinical efficiency, improved patient safety, and measurable outcomes.

## CONCLUSION

The development of an informatics strategy for oncology must start by looking at systems in terms of meeting business needs and goals. Table 1 illustrates some key elements to include in such a strategy.

**Table 1:** Elements of an Oncology Informatics Strategy.

Business Goals
Patient Safety
Economics -Tracking and Management of Costs and Revenue
Metrics for Measuring Quality of Cancer Care, Outcomes, and Performance
Evidence-based Medicine

System Requirements
Adaptable for Future Technologies in Diagnostics and Treatment
Configurable for Patient Population Factors
Designed for System Usability and Interoperability

The first step is to focus on business goals that guarantee:

- Patient safety is ensured with a concise summary of the history of care and with validation and alerts for dosages, drug-to-drug interactions, and allergy or co-morbidity risks.
- Metrics are collected and are able to demonstrate treatment performance and outcomes [18].
- Treatment decisions are guided by standards of care that are set according to collected data and evidence of treatment efficacy and toxicities.
- Diagnostic orders are based on efficacy of the test, technique, or procedure and diagnosis decisions are supported with evidence.
- Physicians and Administrators are enabled to make decisions that are informed by costs and revenue for treatment and diagnostic procedures.

The next step in the strategic process is to define requirements for systems that will help to meet these business goals and that will provide for flexibility, such that:

- Regulatory changes and new technologies in diagnostics and treatments can be incorporated rapidly.
- Treatments and diagnostics can be aligned with patient population and availability.
- Functional use of systems fit with clinician thought-flow.
- Systems work together as a whole to facilitate, and not impede, clinician workflow.

When evaluating oncology software on the market, it is equally as important to understand a vendor’s strategy and costs for licensing, customisation and enhancements, training and implementation, warranties, and support policies, as well as to understand the associated hardware and network requirements and the vendor’s methods for quality assurance. Combining a full evaluation of implementation and support costs and policies with an analysis of how well the system meets the business goals and the needs of stakeholders will contribute to a successful informatics strategy. Communicating and promoting this strategy to software vendors is one way that the oncology industry can enlist the informatics industry to help achieve its goals.

## References

[1] Institute of Medicine of the National Academies (2007). Rewarding Provider Performance: Aligning Incentives in Medicare [Online].

[2] (January 2005). Medicare "pay for performance (P4P)" initiatives [Online].

[3] Trisolini M, Pope G, Kautter J Medicare Physician Group Practices : Innovations in Quality and Efficiency.

[4] Tunis S, Korn A, Ommaya A The Role of Purchasers and Payers in the Clinical Research Enterprise: Workshop Summary.

[5] Cancer Biomedical Informatics Grid (caBIG) [Online].

[6] Edwards J, Handler T, Hieb B (2007). Hype Cycle for Healthcare Provider Applications and Systems.

[7] caBIG™ and Molecular Medicine [Online].

[8] Nass SJ, Moses HL (2007). Cancer Biomarkers: The Promises and Challenges of Improving Detection and Treatment.

[9] (2007). Hype Cycle for Healthcare Provider Technologies and Standards. Gartner Inc.

[10] Rishel W (2006). Tutorial: The Semantic Web for Healthcare.

[11] Oak MR (2007). A review on barriers to implementing health informatics in developing countries. J Health Informatics in Developing Countries.

[12] National Comprehensive Cancer Network Inc (2007). Clinical Practice Guidelines in Oncology (Multiple languages) [Online].

[13] National Comprehensive Cancer Network Inc (2007). Clinical Practice Guidelines in Oncology [Online].

[14] World Health Organization - Regional Office for the Western Pacific. Publications and documents [Online].

[15] Taipei Branch of Bureau of National Health Insurance (2006). Vision and Policy [Online].

[16] Department of Health (2008). Quality and Outcomes Framework (QOF) [Online].

[17] National Center for Biotechnology Information (NCBI) PubMed Database [Online].

[18] Eden J, Simone JV (2005). Assessing the Quality of Cancer Care: An Approach to Measurement in Georgia.

